# MEASURING MULTIGENERATIONAL NEIGHBORHOODS

**DOI:** 10.1093/geroni/igad104.2638

**Published:** 2023-12-21

**Authors:** Martin Hyde, Elizabeth Evans

**Affiliations:** Swansea University, Swansea, Wales, United Kingdom; Swansea University, Swansea, Wales, United Kingdom

## Abstract

There are growing concerns that societies are becoming more generationally divided and that this is having negative social consequences, such as increasing loneliness and social isolation. Residential age segregation as a key driver of this generational divide and there is worrying evidence that levels of residential age segregation have increased in the USA, UK and Europe. However, measures of age segregation have a number of methodological limitations: i) they only compare younger and older age groups and therefore overlook the wider generational mix in the neighbourhood; ii) different measures of young/older age groups are used in different studies, iii) different geographical scales, e.g. wards, districts, etc., are used in different studies and iv) measures are often at a high level of geographical aggregation, e.g. local authorities. To address these issues we have developed a new measure of multigenerational neighbourhoods, based on an ecological measure used to calculate biodiversity, for all neighbourhoods in England and Wales from 2011-2020. Our analyses shows that i) there is a low correlation between measures of age segregation and neighbourhood multigenerationality and ii) contrary to some findings on age segregation, the vast majority of people in England and Wales live in multigenerational neighbourhoods. However, some areas have seen a drop in multigenerationality, especially those in inner city and costal areas.

